# Responses of *Phaseolus calcaltus* to lime and biochar application in an acid soil

**DOI:** 10.7717/peerj.6346

**Published:** 2019-02-12

**Authors:** Luhua Yao, Xiangyu Yu, Lei Huang, Xuefeng Zhang, Dengke Wang, Xiao Zhao, Yang Li, Zhibin He, Lin Kang, Xiaoting Li, Dan Liu, Qianlin Xiao, Yanjun Guo

**Affiliations:** College of Agronomy and Biotechnology, Southwest University, Chongqing, China

**Keywords:** Growth, Rice bean (*Phaseotus calcaltus*), Soil fertility, Nodulation, Nutrient uptake

## Abstract

**Introduction:**

Rice bean (*Phaseolus calcaltus*), as an annual summer legume, is always subjected to acid soils in tropical to subtropical regions, limiting its growth and nodulation. However, little is known about its responses to lime and biochar addition, the two in improving soil fertility in acid soils.

**Materials and Methods:**

In the current study, a pot experiment was conducted using rice bean on a sandy yellow soil (Orthic Acrisol) with a pH of 5.5. The experiment included three lime rates (0, 0.75 and 1.5 g kg^−1^) and three biochar rates (0, 5 and 10 g kg^−1^). The biochar was produced from aboveground parts of *Solanum tuberosum* using a home-made device with temperature of pyrolysis about 500 °C.

**Results and Discussion:**

The results indicated that both lime and biochar could reduce soil exchange Al concentration, increase soil pH and the contents of soil microbial biomass carbon and microbial biomass nitrogen, and enhance urease and dehydrogenase activities, benefiting *P. calcaltus* growth and nodulation in acid soils. Lime application did decrease the concentrations of soil available phosphorus (AP) and alkali dispelled nitrogen (AN), whereas biochar application increased the concentrations of soil AP, AN and available potassium (AK). However, sole biochar application could not achieve as much yield increase as lime application did. High lime rate (1.5 g lime kg^−1^) incorporated with low biochar rate (5 g biochar kg^−1^) could obtain higher shoot biomass, nutrient uptake, and nodule number when compared with high lime rate and high biochar rate.

**Conclusion:**

Lime incorporated with biochar application could achieve optimum improvement for *P. calcaltus* growing in acid soils when compared with sole lime or biochar addition.

## Introduction

Green manure, as a biological tool, has been shown to be efficient in improving the soil quality, particularly the legume plants due to their higher nitrogen (N) contents and biological N fixation abilities (Thorup-Kristensen, Magid & Jensen, 2003). Fast-growing legume green manure crops have a tremendous potential in fixing the atmospheric N_2_, and act as substitute for fertilizer N in wetland rice (Zhan, Li & Cheng, 2007). The green manure legume decomposition releases considerable amounts of mineral N and significant improvements were observed in the microbial biomass, dehydrogenase activity, and bacterial population in a green-manured rice soil (Hamonts et al., 2017). Green manure legumes also could reduce soil bulk density and enhance total porosity, macropores and large mesopores (Sultani et al., 2007).

Rice bean (*Phaseolus calcaltus* Roxb) is an annual legume that can be used as summer green manure (Dahipahle et al., 2017; Tomooka, 1971), mainly distributed in subtropical regions of China. Rice bean has strong branching and nodulation abilities, with shoot biomass reaching about 8,000 kg DM per hectare (Du et al., 2016). However, acid soils are also widely distributed in tropical and subtropical regions (Edmeades & Ridley, 2003), and they negatively influence the growth of green manure legume crops and their abilities of biological nitrogen fixation, resulting in lower yield and nutrient uptake, and thus low efficiency in improving soil quality (Sharifi et al., 2014; Yang et al., 2011). Up to date, the growth of green manure legume in acid soil has been paid less attention when compared with other legume crops such as *Medicago alfalfa* and *Glycine max*.

In acid soils, low pH and high Al^3+^ concentration are two main factors limiting legume plant growth. They inhibit the growth of Rhizobia, reduce the affinity between Rhizobia and plant roots, and inhibit nodulation and nitrogen fixation (Bhattacharyya, Pal & Basumajumdar, 2009; Scandalios, 1993). In the presence of 50 µM Al in a culture medium, numbers of *Rhizobium trifolii* declined around subterranean clover roots, and nodules were not formed, according with Whelan & Alexander (1986). A study on five annual pasture legumes in an acidic loamy sand has shown that establishment of nodules was more sensitive to acidity than indicated by relative yields of dry matter, and average nodule weight usually increased at lower pH but nodule number declined with increasing acidity (Evans, Dear & O’Connor, 1990). Low pH and high Al^3+^ concentration also reduce plant nutrients uptake, root growth, and shoot biomass (Haynes & Ludecke, 1981; Khu, Brummer & Monteros, 2012). A study on 14 tropical legume cover crops also has shown that shoot dry weight of cover crops was significantly affected by soil acidity and the maximum shoot dry weight was produced at high pH (Fageria, Baligar & Li, 2009). Furthermore, low soil available phosphorus (AP) concentrations are always produced in acid soils, limiting legume plant growth (Fageria, Zimmermann & Baligar, 1995).

Liming is a common worldwide agricultural practice used for increasing productivity in acid agricultural soils (Paradelo, Virto & Chenu, 2015). In general, soil fertility and crop yield increase significantly by liming acid soils with pH less than 5.5 (MacGregor, Pearson & Adams, 1968). On an acid siliceous sand in the South East of South Australia, soil pH was significantly increased to a depth of 12.5 cm by the application of 4 t ha^−1^ of lime, increasing the seed yields of subterranean clover (Hodge & Lewis, 1994). Lime application significantly reduced the soil exchange Al concentration (Martini & Mutters, 1985), increased the root growth, nodulation, herbage yield, and contents of crude protein and calcium of alfalfa (Grewal & Williams, 2003). In an acid soil, nodulation and N content of white clover increased significantly with increasing lime applications, whereas Al contents of shoots and roots decreased with increasing lime rates, and there was a highly significant negative correlation between relative yield and Al content of white clover tops (Haynes & Ludecke, 1981). Deficiency of available phosphorus (AP) due to high fixation by iron (Fe) and aluminum oxides in acid soils could also be overcome by liming (Patiram, 2007). However, on acid soils, increased levels of lime tend to reduce uptake of P, zinc (Zn), copper (Cu), manganese (Mn), and Fe in *Oryza sativa*, *Triticum aestivum*, *Phaseolus vulgaris*, and *Zea mays* (Fageria, Zimmermann & Baligar, 1995). Lime also induced magnesium stress in corn, limiting soil magnesium and phosphorus availability (Grove & Sumner, 1985). Furthermore, Nunes et al. (2017) reported that excessive lime decreased specific surface area, anisotropy degree and the connectivity of the soil pore system, and finally altered the soil physicochemical properties and impacted soil structure..

It has been shown that adding organic amendments to acid soils, such as biochar and biosolids can enhance the soil fertility similar to lime application (Sarma et al., 2017). Biochar is produced by pyrolysis of biomass under 300–500 °C and is used as a soil amendment (Spokas, Novak & Venterea, 2012). In an Acrorthox soil, biochar increased the pH, reduced the amount of the total active aluminum and exchangeable Al^3+^, increased the content of available potassium (AK), AP, and organic carbon, and improved cabbage growth (Lin et al., 2018). In acid soils, biochar addition could increase cation exchange capacity (CEC) and P content, and reduce the availability of toxic metals, improving plant productivity (Chintala et al., 2014). Biochar can also affect soil microbial enzymatic activities (Ameloot et al., 2015), which might be positively correlated with the increase of soil C/N ratio in biochar added soils (Jiang et al., 2016). In a moderately acidic Inceptisol, Legay et al. (2017) reported that the alleviation of nutrient stress was probably the main factor contributing to the increased maize biomass production upon biochar addition, whereas the acidity stress alleviation was not the mechanism of biochar effects on soil fertility. Teutscherova et al. (2017) reported that biochar could result in more pronounced changes in N cycle than lime application which could be of especially high interest in intensively managed soils with high N inputs. These results suggested that biochar application might be an alternative way besides traditional liming in improving acid soil fertility for green manure legume growth.

In order to evaluate the efficiency of biochar from *Solanum tuberosum* and the lime in improving growth of rice bean in acid soils, in the current study, a pot experiment was conducted using rice bean, in a sandy yellow soil (Orthic Acrisol) with a pH of 5.5. Soils were amended with different lime and biochar rates. We hypothesized that biochar addition might be an alternative in improving rice bean growth in acid soils. The following three aspects were mainly addressed: (1) soil chemical properties; (2) soil biological and biochemical properties (microbial biomass carbon, microbial biomass nitrogen, enzymes, and nitrogen fixation ability); (3) plant growth and nutrient uptake.

## Materials and Methods

### Soil sampling and characterization

The soil (Orthic Acrisol) used in this study was collected from arable land, located in Mountain Jigong (E106.42; N29.83°), Chongqing, China. During the last ten years, corn and sweet potato are the main crops in the arable land. The climate in this area is subtropical monsoon humid climate with mean annual precipitation reaching 1133.7 mm and mean annual temperature reaching 18 °C (Chongqing Meteorological Bureau, 2012–2016). Soils were sampled using a shovel at the depth of 0–20 cm, air-dried, and sieved through a 2 mm sieve. The soil was then sterilized at 121 °C in autoclave for 25 min for two times to completely get rid of soil microbes. The soils were analyzed for soil parameters according to the methods mentioned in following Soil sampling and analysis. The concentrations of total organic carbon (TOC), total nitrogen (TN), total phosphorus (TP) and total potassium (TK) in the tested soils were 22.83, 0.79, 0.56 and 18.33 g kg^−1^ respectively. The concentrations of alkali dispelled N (AN), available phosphorus (AP, Bray), available potassium (AK) and exchangeable aluminum (Al^3+^) were 100.12, 20.16, 112.5 and 1126.82 mg kg^−1^ (4.17 cmol kg^−1^), respectively. The soil cation exchange capacity (CEC) was 8.54 cmol kg^−1^. The Al saturation was 48.83%. The soil pH was 5.5 (soil: water = 1:2.5).

### Biochar preparation

The biochar was produced using a home-made pyrolysis device (small metal pot with plant materials inside was put upside down in a big metal bucket which was used to fill fuels to heat the small bucket). Aboveground parts of *Solanum tuberosum*, which normally have been discarded after harvesting the tubers, were collected, air dried, pyrolysed at a highest treatment temperature of 550 °C for 4–5 h, and then ground to pass a 2 mm sieve. The pH of the biochar was 9.89 and measured using a biochar to deionized water mixture (1: 5) followed by shaking and an equilibration time of 30 min before measurement with a pH meter (Bao, 2005). The biochar (0.100 g) was extracted with deionized water at 25 °C and the filtrates were used to analyze AP (Mo-Sb colorimetric procedure), AK (flame photometer method) and AN (alkaline diffusion method) (Bao, 2005). The concentrations of AN, AP and AK were 11.66, 420.95 and 49.33 mg kg^−1^, respectively. Total organic carbon (TOC) in biochar was determined by oxidation with potassium dichromate in a concentrated sulfuric acid medium and the excess dichromate was measured using Mohr’s salt (NH_4_)_2_Fe(SO_4_)_2_ ⋅ 6H_2_O (Yeomans & Bremner, 1988). TN was analyzed by Kjeldahl method, TP was measured using Mo-Sb colorimetric procedure (Bao, 2005), TK was measured using flame photometry (Bao, 2005). The concentrations of TOC, TN, TP and TK were 753.65, 2.60, 1.37 and 238.75 g kg^−1^, respectively.

### Rhizobia inoculant preparation

The tested Rhizobia were separated from root nodules of *Phaseolus calcaltus* in field. They were purified and propagated using YMA medium (Yeast Mannitol Agar). The inoculants were propagated using YMA medium without agar (Bhargava et al., 2016).

### Experimental design

The experiment was a two-way random design with three biochar rates, 0 g kg^−1^, 5 g kg^−1^ (equaling 5,659 kg ha^−1^) and 10 g kg^−1^ (equaling 11,318 kg ha^−1^) and three lime rates, 0 g kg^−1^, 0.75 g kg^−1^ (equaling 848 kg ha^−1^) and 1.5 g kg^−1^ (equaling 1,697 kg ha^−1^), with four replications. The lime and biochar were mixed thoroughly with soils. In a pre-experiment, liming the tested soil with 0.75 g lime kg^−1^ and 1.5 g lime kg^−1^ could obtain pH of 6.0 and 6.4, respectively. About 2.0 kg soil was filled into a plastic bag, then put in a pot (15 cm × 20 cm), and taken to field water capacity for 15 days before sowing. The soil field water capacity was calculated previously.

Seeds of *Phaseolus calcaltus* were sterilized in 3% H_2_O_2_, washed three times, then planted in pot with 6 seeds in each pot. The pots were watered with 20 mL Rhizobia solution for three times during the first three weeks after seeding. The relative soil water contents were kept around 70–80% of the field water capacity through adding water. The pots were placed in a glass greenhouse with temperature ranging from 20 °C to 25 °C, and relative humidity ranging from 65% to 75%. One week after seed sprouting, three seedlings were kept in each pot.

### Harvesting and plant biomass characterization

Two months after seed sprouting, one day before harvest, leaf photosynthesis parameters (the net photosynthesis rate, transpiration rate, stomatal conductance and internal CO_2_ concentration) were measured by using LI-6400XT Portable Photosynthesis System (Li-Cor, Lincoln, NE, USA). The plants were in their branching stage.

The shoots were harvested first, and then about 200 g of soil samples (without roots) were carefully collected at a depth of 5–10 cm from the pot. After soil sampling, the remaining soils with roots were carefully put into a big basin filled with tap water, shaken carefully, until the roots were separated from the soil. The roots were collected on a 1 mm sieve and cleaned free of soils. The soil solutions in the basin were also sieved (1 mm) to collect the abscised nodules and fine roots. Nodules were separated manually with tweezers from the roots, and the nodule numbers were counted. The shoot biomass was also washed two times in clean tap water. Then, the plants, roots and nodules were dried at 75 °C for 48 h, and weighed (Bao, 2005). The plants were ground to pass 2 mm sieve for chemical analysis.

About 0.4 g plant sample was digested in 5 ml 18.4 M H_2_SO_4_ until the solution color turned to transparent, then nutrients were analyzed by methods of Bao (2005). Then, TN was analyzed by Kjeldahl method, TP was measured using the Mo-Sb colorimetric procedure (Bao, 2005), TK was measured using flame photometry (Bao, 2005). The concentration of plant aluminum was measured by Eriochrome cyanine R spectrophotometric method (Dougan & Wilson, 1974).

### Soil sampling and analysis

Dried soils (1.00 g) were digested in 5 mL H_2_SO_4_ and then analysed for TN by Kjeldahl method (Bao, 2005). Dried soils (0.2500 g) were melted with NaOH at high temperature to measure TP using the Mo-Sb colorimetric procedure and TK using flame photometry (Bao, 2005). Dried soils (2.000 g) were digested in 10 m L 1 mol L^−1^ NaOH solution for 24 h and titrated with 0.01 mol L^−1^ 1/2 H_2_SO_4_ for alkali dispelled nitrogen (AN) (Bao, 2005). Dried soils (1.000 g) were mixed and shaken in 7 mL 1.0 mol L^−1^ NH_4_F for 30 min, and then the supernatant was analyzed for AP using Mo-Sb colorimetric procedure (Bao, 2005). Dried soils (5.000 g) were extracted by 50 mL 1 mol L^−1^ NH_4_Ac solution for 15 min, and then the supernatant was analyzed for AK using the flame photometer method (Bao, 2005). Soil total organic carbon (TOC) was determined by oxidation with potassium dichromate in a concentrated sulfuric acid medium and the excess dichromate was measured using Mohr’s salt (NH_4_)_2_Fe(SO_4_)_2_ ⋅ 6H_2_O (Yeomans & Bremner, 1988).

Dried soils (0.200 g) were extracted with 1 mol L^−1^ KCl and exchangeable Al concentration was determined by Eriochrome cyanine R spectrophotometric method (Dougan & Wilson, 1974). Dried soils (2.00 g) were extracted with 0.1 mol L^−1^ BaCl_2_ and then 0.01 mol L^−1^ (1/2 MgSO_4_), and then measured the amount of added cation that was retained (Bao, 2005). Soil pH value was determined in a soil: water solution (1:2.5) using a pH meter (Bao, 2005) .

Urease activity was determined as the NH_4_ released in the hydrolysis reaction of urea (Nannipieri et al., 1982). Triphenyltetrazolium chloride (TTC) was used as the substrate (1:1 soil: solution, w/v) to determine dehydrogenase activity (Casida, Klein & Santoro, 1964), which was expressed as µg TTF per g soil within 24 h.

The soil microbial biomass carbon (MBC) and microbial biomass nitrogen (MBN) was measured by the fumigation-extraction procedure (Brookes et al., 1985). Ten grams of fresh soil samples fumigated with chloroform and non-fumigated were extracted with 50 ml of 0.5 mol L^−1^ K_2_SO_4_ separately. TOC and TN in the subsequent filtrates were measured by the methods used for soil samples as mentioned above.

MBC = (A − B) ÷ 0.38

A: extractable organic C in fumigated.

B: extractable organic C non-fumigated soil.

0.38: The conversion factor for microbial biomass carbon.

MBN = (C − D) ÷ 0.45

C: extractable organic N in fumigated.

D: extractable organic N non-fumigated soil.

0.45: The conversion factor for microbial biomass nitrogen

### Statistical analysis

Data were subjected to a two-way ANOVA analysis using SPSS version 17 software (SPSS, Inc., Chicago, IL, USA), to analyze the effects of lime and biochar and their interactions on soil and plant parameters. One way ANOVA analysis was further applied to analyze the effects of biochar and lime application on soil and plant parameters. Significance was tested according to the least significant difference (L.S.D) test at *P* = 0.05. The relationship between the soil pH and the plant growth, nutrient concentration and uptake, and soil chemical and biological parameters was fitted to simple linear model or quadratic model using SigmaPlot 10.0 (Systat Software, Inc., Chicago, IL, USA).

## Results

### Effect of treatments on soil chemical properties

Soil pH and the concentrations of soil exchangeable Al and available nutrients were significantly influenced by lime and biochar applications and their interactions (Table 1). Lime application significantly increased the pH from 5.49 at 0 g kg^−1^ (L0) to 6.30 at 1.5 g lime kg^−1^ (L2) without biochar application. Within each lime rate, biochar application, particularly at 1.5 g kg^−1^, significantly increased the pH. The concentrations of soil exchangeable Al reduced significantly with increased lime rates and biochar rates. The concentrations of AP increased at B2 under L0 condition, at B1 and B2 under L1 condition, and at B1 under L2 condition. Biochar application significantly increased the concentrations of AK and AN and overall higher concentrations of AK and AN were observed at B2 than B1. Without biochar addition, the concentrations of soil AP at L0 were significantly higher than those at L1 and L2; while the concentrations of soil AN and AK changed insignificantly with lime application.

**Table 1:** Effects of lime and biochar application on soil pH and concentrations of soil exchangeable Al and available nutrients.

**Lime rates**	**Biochar rates**	**pH (soil:water = 1:2.5)**	**Exchangeable Al (cmol kg^−1^)**	**AP(mg kg^−1^)**	**AK(mg kg^−1^)**	**AN(mg kg^−1^)**
L0	B0	5.49(0.01)^f^	3.39(0.07)^a^	39.00(0.23)^b^	141.25(5.54)^d^	57.75(1.75)^cde^
	B1	5.54(0.03)^ef^	2.64(0.04)^b^	39.09(0.30)^b^	200.00(4.56)^b^	89.25(13.82)^a^
	B2	5.84(0.09)^c^	2.64(0.05)^b^	41.64(0.25)^a^	247.50(3.23)^a^	84.00(4.95)^ab^
L1	B0	5.67(0.03)^de^	2.14(0.03)^c^	37.68(0.17)^c^	151.25(8.26)^d^	42.00(2.86)^e^
	B1	5.75(0.02)^cd^	1.83(0.03)^d^	35.45(0.16)^e^	200.00(4.56)^b^	70.00(2.86)^bc^
	B2	5.90(0.09)^c^	1.64(0.02)^e^	36.74(0.08)^d^	210.00(2.04)^b^	85.75(4.40)^ab^
L2	B0	6.30(0.05)^b^	1.04(0.02)^f^	32.31(0.25)^f^	140.00(3.54)^d^	50.75(3.35)^de^
	B1	6.29(0.05)^b^	0.75(0.03)^g^	36.05(0.23)^e^	183.75(4.27)^c^	61.25(3.35)^cd^
	B2	6.55(0.06)^a^	0.33(0.02)^h^	31.78(0.19)^f^	210.00(2.04)^b^	73.50(2.02)^abc^
		ANOVA analysis (*F* value)				
Lime (L)		156.48^***^	2506.66^***^	687.389^***^	11.93^***^	5.92^**^
Biochar (B)		21.97^***^	235.67^***^	4.88^*^	223.87^***^	24.97^***^
L * B		0.67ns	15.31^***^	93.05^***^	8.11^***^	2.03ns

**Notes.**

The number in parentheses is the standard error. Same below.

L0, L1 and L2 represented 0 g kg^−1^, 0.75 g lime kg^−1^ and 1.5 g lime kg^−1^, respectively; B0, B1 and B2 represented 0 g kg^−1^, 5 g biochar kg^−1^ and 10 g biochar kg^−1^, respectively. AP, available phosphorus; AK, available potassium; AN, alkali dispelled nitrogen.

Different lowercase letters followed after data represented significance at *P* < 0.05 according to the least significant difference test.

**Figure 1:**
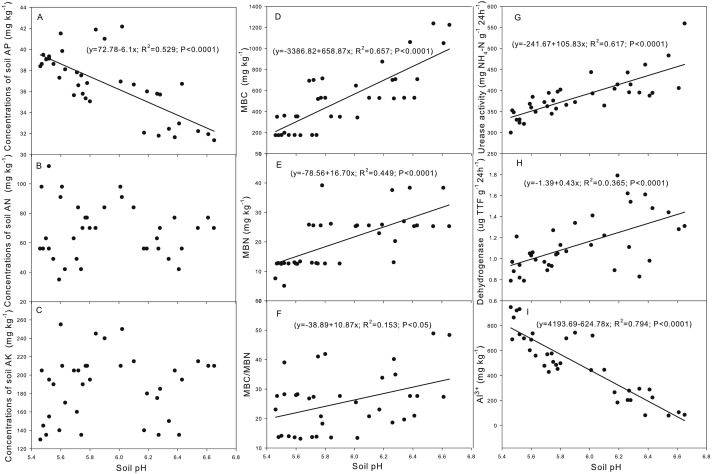
Correlation between soil pH and the soil properties. (A) AP (available phosphorus); (B) AN (alkali dispelled N); (C) AK (available potassium); (D) MBC (microbial biomass carbon); (E) MBN (microbial biomass nitrogen); (F) MBC/MBN (microbial biomass carbon / microbial biomass nitrogen); (G) Urease activity; (H) Dehydrogenase activity; (I) Al3+(exchangeable Al).

Correlation analysis indicated that the concentration of AP was negatively correlated with soil pH (*y* = 72.78 − 6.10*x*; *R*^2^ = 0.529; *P* < 0.0001) (Fig. 1A). Also, a significant negative correlation was observed between soil pH and the concentration of soil exchangeable Al concentration (Fig. 1I).

### Effect of treatments on soil biological and biochemical properties

The contents of MBC and MBN and soil enzyme activities were significantly influenced by lime and biochar applications and their interactions (Table 2). The MBC and MBN increased with increased biochar rates and lime rates (*P* < 0.05). However, the ratio of MBC/MBN changed insignificantly, excepting a higher ratio at B2 under L1 condition. Biochar application increased the urease activity under all lime rates, with significance only at B2. The urease activities also increased with increased lime rates (*P* > 0.05). Biochar application increased the dehydrogenase activity under all lime rates except for B1 at L0, with significant changes being observed only at L2. Lime application showed no significant influence on dehydrogenase activity at B0.

**Table 2:** Effects of lime and biochar application on soil microbial carbon, nitrogen and soil enzyme activities.

**Lime rates**	**Biochar rates**	**MBC (mg kg^−1^)**	**MBN (mg kg^−1^)**	**MBC/MBN**	**Urease activity (mg NH_4_-N g^−1^ 24 h)**	**Dehydrogenase activity (ug TTF g^−1^ 24 h)**
L0	B0	181.02(5.52)^f^	9.50(1.90)^e^	22.45(5.92)^bc^	325.48(10.03)^e^	0.96(0.09)^d^
	B1	310.28(44.57)^e^	12.66(0.05)^de^	24.51(3.52)^abc^	347.38(14.21)^de^	0.91(0.06)^d^
	B2	349.86(2.55)^e^	19.26(3.81)^cd^	20.62(4.13)^bc^	372.74(7.26)^bcd^	1.21(0.10)^bc^
L1	B0	176.28(0.46)^f^	13.02(0.12)^de^	13.54(0.15)^c^	356.24(5.51)^cde^	0.97(0.04)^d^
	B1	568.03(41.53)^cd^	19.19(3.77)^cd^	32.60(5.26)^ab^	376.81(9.38)^bcd^	1.10(0.06)^cd^
	B2	647.52(41.79)^c^	28.92(3.42)^ab^	22.95(2.10)^bc^	399.83(16.40)^bc^	1.09(0.06)^d^
L2	B0	528.38(1.30)^d^	22.07(3.12)^bce^	25.94(4.78) ^abc^	395.68(3.33)^bc^	0.95(0.06)^d^
	B1	747.01(42.53)^b^	27.32(3.66)^abc^	28.74(3.74)^ab^	416.43(9,97)^b^	1.61(0.07)^a^
	B2	1142.83(50.87)^a^	31.85(3.77)^a^	38.05(6.10)^a^	477.43(31.79)^a^	1.41(0.08)^ab^
		ANOVA analysis (*F* value)				
Lime (L)		194.25^***^	14.62^***^	3.49^*^	24.59^***^	16.66^***^
Biochar (B)		120.58^***^	11.69^***^	2.86ns	12.27^***^	164.32^***^
L*B		16.32^***^	0.40 s	2.12^*^	0.72ns	7.64^***^

**Notes.**

L0, L1 and L2 represented 0 g kg^−1^, 0.75 g lime kg^−1^ and 1.5 g lime kg^−1^, respectively; B0, B1 and B2 represented 0 g kg^−1^, 5 g biochar kg^−1^ and 10 g biochar kg^−1^, respectively.

MBC Microbial biomass carbon MBN microbial biomass nitrogen

Different lowercase letters followed after data represented significance at *P* < 0.05 according to the least significant difference test.

Correlation analysis indicated that the MBC, MBN, MBC/MBN, and the activities of urease and dehydrogenase were positively correlated with soil pH (Figs. 1D–1H).

### Leaf photosynthesis

There was no significant interaction between lime and biochar applications on parameters of leaf photosynthesis (Table 3). Lime application significantly influenced all parameters of leaf photosynthesis, whereas biochar application only influenced the net photosynthesis rate (Table 3). The net photosynthesis rate, transpiration rate, stomatal conductance and internal CO_2_ concentration of plants in limed soils were significantly higher than those in un-limed soils. Biochar application increased the net photosynthesis rate at B2 under L0 and L2, increased transpiration rate at B2 under L2, with the other photosynthesis parameters unchanged.

**Table 3:** Effect of lime and biochar applications on leaf photosynthesis.

**Lime rates**	**Biochar rates**	**Net photosynthesis rate µmol m^−2^ s^−1^**	**Transpiration rate mmol m^−2^ s^−1^**	**Stomatal conductance mol m^−2^ s^−1^**	**Internal CO_2_ concentration Mmol CO_2_ mol^−1^**
L0	B0	5.71(0.49)^e^	2.78(0.31)^bc^	0.18(0.04)^c^	311.75(9.45)^bc^
	B1	5.63(0.35)^e^	2.22(0.23)^c^	0.15(0.02)^c^	304.50(5.11)^c^
	B2	6.86(0.51)^d^	2.83(0.20)^bc^	0.15(0.02)^c^	314.25(4.96)^bc^
L1	B0	9.32(0.38)^c^	3.82(0.11)^bc^	0.27(0.01)^bc^	335.25(8.68)^ab^
	B1	9.32(0.41)^c^	4.34(0.59)^ab^	0.25(0.04)^c^	337.75(10.08)^ab^
	B2	10.16(0.22)^bc^	3.43(0.24)^bc^	0.26(0.03)^c^	328.50(7.50)^bc^
L2	B0	10.80(0.17)^b^	3.29(1.01)^bc^	0.43(0.11)^ab^	338.75(8.38)^ab^
	B1	10.90(0.52)^b^	3.50(0.32)^bc^	0.32(0.07)^bc^	323.75(9.01)^bc^
	B2	12.60(0.35)^a^	5.41(0.83)^a^	0.53(0.07)^a^	358.67(10.96)^a^
		ANOVA analysis (*F* value)			
Lime (L)		143.35^***^	6.93^**^	18.69^***^	10.59^***^
Biochar (B)		10.23^***^	1.21ns	1.41ns	1.47ns
L*B		0.46ns	2.53ns	1.15ns	1.75ns

**Notes.**

L0, L1 and L2 represented 0 g kg^−1^, 0.75 g lime kg^−1^ and 1.5 g lime kg^−1^, respectively; B0, B1 and B2 represented 0 g kg^−1^, 5 g biochar kg^−1^ and 10 g biochar kg^−1^, respectively.

Different lowercase letters followed after data represented significance at *P* < 0.05 according to the least significant difference test.

### Plant biomass and nodulation

**Figure 2:**
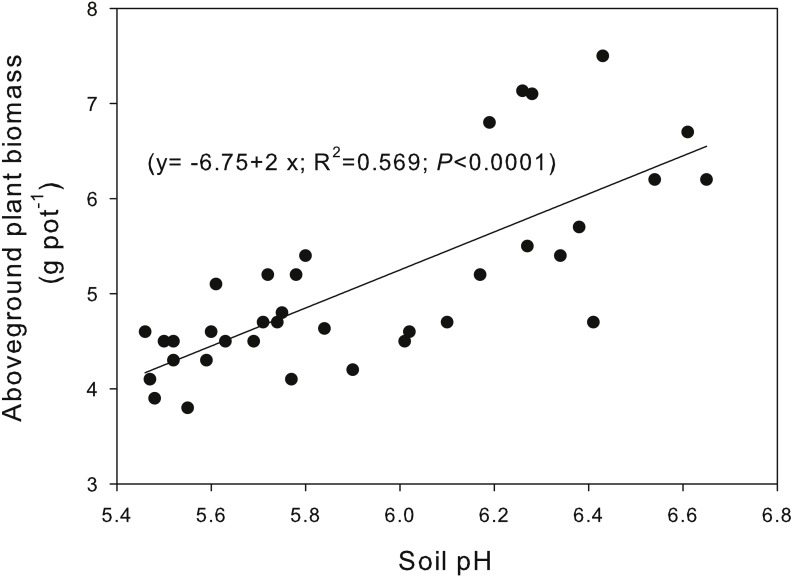
Relationship between the plant aboveground biomass of *Phaseolus Calcaltus* (g pot^−1^) and the soil pH.

Both lime and biochar applications and their interactions significantly influenced the shoot and root biomass, root/shoot ratio, and the total biomass, excluding the insignificant influence of biochar on root biomass (Table 4). Within each lime rate, biochar application had no significant influence on shoot biomass, root/shoot ratio and total biomass under L0 and L1 conditions, whereas significantly increased the shoot biomass and total biomass and reduced root/ratio under L2 condition, excepting for insignificant changes of total biomass at B2. Without biochar addition, the shoot biomass and root biomass and total biomass at L2 were significantly higher than those at L0, while the root/shoot ratio changed insignificantly. Correlation analysis indicated that the shoot biomass was positively correlated with soil pH (*y* =  − 6.75 + 2*x*; *R*^2^ = 0.569; *P* < 0.0001) (Fig. 2).

**Table 4:** Effect of lime and biochar applications on plant growth.

**Lime rates**	**Biochar rates**	**Aboveground biomass (g pot^−1^)**	**Belowground biomass (g pot^−1^)**	**Root/shoot**	**Total biomass (g pot^−1^)**
L0	B0	4.33(0.15)^d^	3.31(0.08)^c^	0.77(0.02)^a^	7.64(0.21)^d^
	B1	4.38(0.28)^d^	3.34(0.15)^bc^	0.77(0.02)^a^	7.71(0.43)^d^
	B2	4.51(0.10)^d^	3.29(0.16)^c^	0.73(0.04)^a^	7.80(0.17)^d^
L1	B0	4.55(0.10)^d^	3.44(0.03)^abc^	0.76(0.01)^a^	7.99(0.12)^d^
	B1	4.70(0.27)^cd^	3.44(0.15)^abc^	0.73(0.02)^a^	8.14(0.42)^d^
	B2	4.90(0.18)^cd^	3.55(0.07)^abc^	0.73(0.02)^a^	8.45(0.24)^cd^
L2	B0	5.20(0.18)^c^	3.86(0.17)^a^	0.74(0.02)^a^	9.06(0.33)^bc^
	B1	7.13(0.14)^a^	3.81(0.24)^ab^	0.54(0.04)^b^	10.94(0.21)^a^
	B2	6.20(0.20)^b^	3.60(0.19)^abc^	0.58(0.02)^b^	9.80(0.36)^b^
		ANOVA analysis (*F* value)			
Lime (L)		74.96^***^	6.59^**^	25.51^***^	47.00^***^
Biochar (B)		11.23^***^	0.11ns	8.49^***^	4.43^**^
L*B		7.95^***^	0.46ns	4.85^**^	3.35^**^

**Notes.**

L0, L1 and L2 represented 0 g kg^−1^, 0.75 g lime kg^−1^ and 1.5 g lime kg^−1^, respectively; B0, B1 and B2 represented 0 g kg^−1^, 5 g biochar kg^−1^ and 10 g biochar kg^−1^, respectively.

Different lowercase letters followed after data represented significance at *P* < 0.05 according to the least significant difference test.

Lime significantly increased the nodule number, dry weight and mean nodule weight (Table 5). The biochar application had no significant influence on nodulation ability, excepting for a trend in increasing the nodule number and dry weight and the mean nodule weight at B2 under L0 and at B1 and B2 under L1 and L2 conditions (*P* > 0.05). The plant failed forming nodule at B0 and B1 under L0 conditions.

**Table 5:** Effects of lime and biochar applications on nodulation.

**Lime rates**	**Biochar rates**	**Nodule**		
		**Number per pot**	**Dry weight per pot (g)**	**Mean nodule weight (g)**
L0	B0	0.00(0.00)^c^	0.00(0.00)^c^	0.00(0.00)^b^
	B1	0.00(0.00)^c^	0.00(0.00)^c^	0.00(0.00)^b^
	B2	27.50(18.87)^bc^	1.40(1.09)^c^	0.23(0.14)^ab^
L1	B0	37.50(22.50)^bc^	1.95(1.13)^bc^	0.27(0.16)^ab^
	B1	82.50(44.98)^abc^	3.03(1.43)^abc^	0.30(0.10)^a^
	B2	147.50(33.26)^a^	5.43(0.92)^a^	0.38(0.03)^a^
L2	B0	57.50(29.55)^abc^	2.28(1.01)^abc^	0.32(0.11)^b^
	B1	157.50(58.36)^a^	4.93(1.67)^ab^	0.24(0.08)^ab^
	B2	122.50(42.11)^ab^	4.48(1.50)^ab^	0.37(0.04)^a^
		ANOVA analysis (*F* value)		
Lime (L)		7.89^**^	8.23^**^	6.71^**^
Biochar (B)		3.25ns	3.29ns	2.29ns
L*B		1.01ns	0.65ns	0.32ns

**Notes.**

L0, L1 and L2 represented 0 g kg^−1^, 0.75 g lime kg^−1^ and 1.5 g lime kg^−1^, respectively; B0, B1 and B2 represented 0 g kg^−1^,5 g biochar kg^−1^ and 10 g biochar kg^−1^, respectively.

Different lowercase letters followed after data represented significance at *P* < 0.05 according to the least significant difference test.

### Plant nutrients and aluminum uptake

Lime application significantly influenced the concentrations of TN and Al in plants, whereas biochar significantly influenced the concentrations of TP and Al, and there existed significant interactions between lime and biochar (Table 6). Biochar had no significant influence on TN and TK at any lime rate, increased TP at B2 under L1 and at B1 under L2, and reduced Al concentration under L0 and L1 but not under L2. Without biochar addition, lime application increased TN only at L2 and had no significant influence on TP and TK. The concentrations of Al significantly reduced with increased lime rates.

**Table 6:** Effect of lime and biochar applications on plant nutrient concentration and uptake.

**Lime rates**	**Biochar rates**	**Nutrient concentration**				**Nutrient uptake**			
		**TN (g kg^−1^)**	**TP (g kg^−1^)**	**TK (g kg^−1^)**	**Al (mg kg^−1^)**	**N (mg pot^−1^)**	**P (mg pot^−1^)**	**K (mg pot^−1^)**	**Al (µg pot^−1^)**
L0	B0	28.15(1.70)^de^	1.98(0.18)^cd^	44.69(2.51)^a^	90.87(0.70)^a^	121.55(8.23)^d^	8.62(0.98)^c^	193.46(13.98)^b^	393.03(14.80)^a^
	B1	26.29(3.62)^e^	1.76(0.15)^d^	42.44(6.46)^a^	53.39(0.86)^bc^	114.83(16.74)^d^	7.76(0.98)^c^	188.65(34.74)^b^	233.66(16.11)^cd^
	B2	35.8(0.36)^cd^	2.52(0.08)^abc^	38.75(2.95)^a^	47.03(3.81)^de^	161.87(5.21)^cd^	11.38(0.60)^bc^	175.30(16.06)^b^	212.51(19.66)^d^
L1	B0	35.47(2.32)^cd^	1.96(0.13)^cd^	38.13(3.90)^a^	56.81(1.81)^b^	161.50(11.74)^cd^	8.95(0.71)^c^	173.18(17.04)^b^	258.40(9.08)^bc^
	B1	43.26(0.72)^c^	2.17(0.17)^bcd^	45.50(2.86)^a^	49.48(1.47)^cd^	203.3(10.05)^c^	10.25(1.13)^bc^	212.11(9.21)^b^	231.73(10.14)^cd^
	B2	36.19(0.80)^cd^	2.84(0.15)^a^	42.06(1.35)^a^	48.50(1.82)^cd^	177.62(9.52)^c^	13.94(1.04)^b^	206.24(11.10)^b^	237.11(8.08)^cd^
L2	B0	64.99(4.93)^ab^	1.89(0.21)^d^	41.50(4.49)^a^	41.49(2.29)^ef^	338.60(31.57)^b^	9.88(1.31)^c^	216.12(25.10)^b^	216.93(18.49)^cd^
	B1	57.45(1.68)^b^	2.55(0.33)^ab^	41.00(1.36)^a^	39.86(0.31)^f^	409.65(12.74)^a^	18.29(2.63)^a^	292.85(14.45)^a^	284.42(6.97)^b^
	B2	66.97(5.14)^a^	1.85(0.10)^d^	46.00(3.02)^a^	41.82(0.86)^ef^	412.22(20.69)^a^	11.39(0.26)^bc^	284.54(17.55)^a^	259.19(9.40)^bc^
		ANOVA analysis (*F* value)							
Lime (L)		104.84^***^	1.68ns	0.07ns	113.72^***^	214.59^***^	7.55^**^	14.78^***^	6.22^**^
Biochar (B)		1.68ns	4.83^*^	0.14ns	79.46^***^	6.27^**^	5.92^**^	3.02^*^	12.91^***^
L*B		3.30^*^	5.52^**^	1.13ns	45.84^***^	2.44ns	6.89^***^	1.62ns	24.86^***^

**Notes.**

L0, L1 and L2 represented 0 g kg^−1^, 0.75 g lime kg^−1^ and 1.5 g lime kg^−1^, respectively; B0, B1 and B2 represented 0 g kg^−1^, 5 g biochar kg^−1^ and 10 g biochar kg^−1^, respectively.

Different lowercase letters followed after data represented significance at *P* < 0.05 according to the least significant difference test.

Both lime and biochar application significantly influenced the uptake of N, P, K and Al (Table 6). Biochar application significantly increased the N uptake at L2 but not at L0 and L1, increased P uptake at both biochar rates under L1 and at B1 under L2, increased K uptake at B1 and B2 under L2. The Al uptake significantly reduced at B1 and B2 under L0, did not changed under L1, and increased at B1 under L2. Without biochar addition, the N uptake at L2 was significantly higher than those at L0 and L1, whereas the P uptake and K uptake changed insignificantly. The Al uptake at L0 was significantly higher than those at L1 and L2.

**Figure 3:**
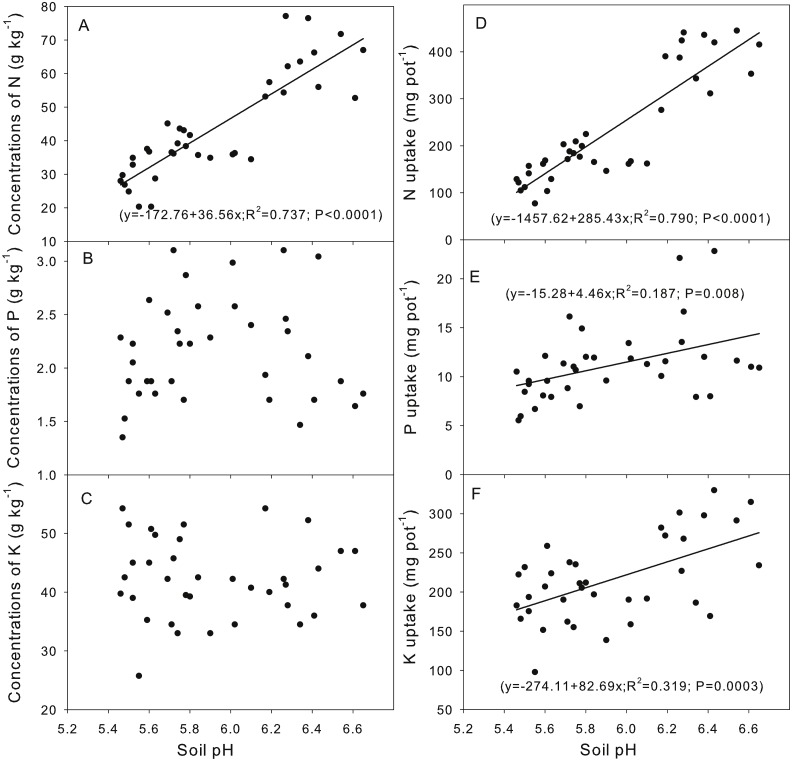
Correlation between the soil pH (A, B, C, D, E, F) and the plant nutrient concentration and uptake. N, nitrogen; P, phosphorus; K, potassium.

**Figure 4:**
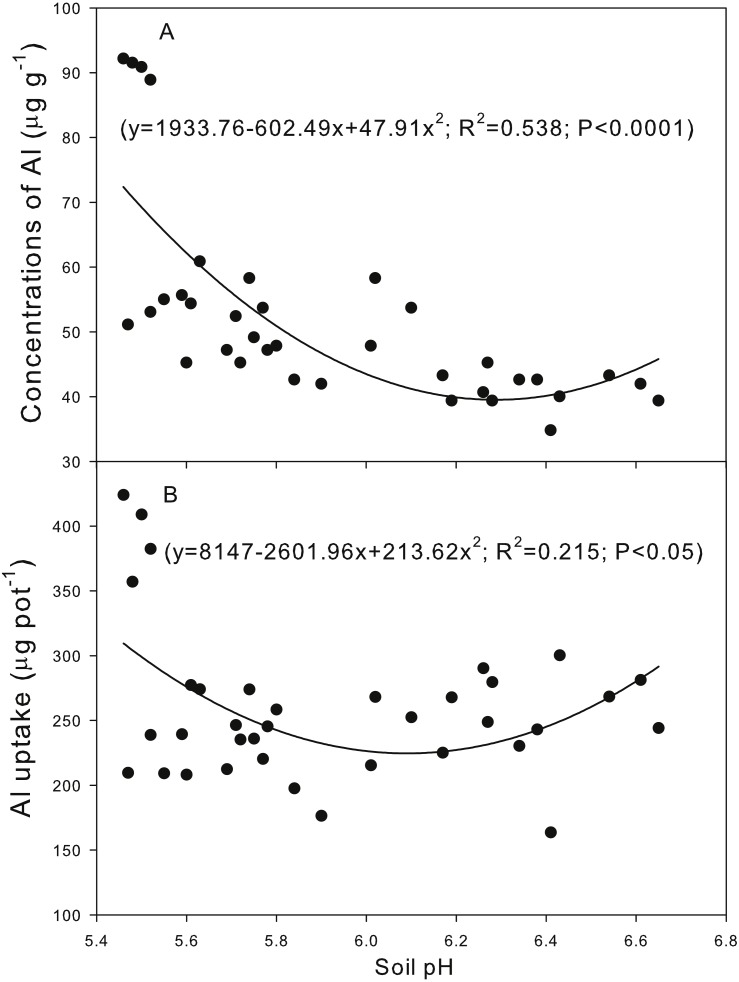
Correlation between the soil pH and the plant aluminum (Al) concentration (A) and Al uptake (B).

Correlation analysis indicated that the soil pH was positively correlated with the concentrations of plant N and the uptake of N, P and K (Fig. 3). A quadratic relationship was observed between the soil pH and the plant Al concentration and Al uptake (Fig. 4).

## Discussion

### Comparison between lime and biochar application in improving acid soil fertility

In acid soils, lime is a common and efficient method in improving soil fertility (Edmeades & Ridley, 2003). It can increase the soil pH, reduce the soil exchangeable Al^3+^ concentration, and correct nutrient deficiencies in acid soils (Akinrinde, 2008). In the current study, when 1.5 g lime kg^−1^ was applied (equaling 1,697 kg lime ha^−1^), the soil pH increased from 5.49 to 6.30 and the soil exchangeable Al concentration reduced by 69.28%, reducing from 3.39 to 1.04 cmol kg^−1^ (Al saturation from 39.69% to 12.18%). However, lime application reduced the concentrations of soil AP, which in turn might negatively influence the plant growth. Correlation analysis also indicated that the concentration of soil AP decreased with increased soil pH and increased with increased soil exchange Al concentration. It has been reported that lime could decrease soil P sorptivity, and thus increase soluble P and the P uptake by plant (Holford, Schweitzer & Crocker, 1994). However, the sudden pH increase after lime application might change the activities of pH-sensitive P cycling enzymes in soils (i,e., phosphatase) and the microbial mineralization of soil organic matter (Robson & Abbot, 2012; Turner, 2010), causing a decrease of soil AP. Margenot, Sommer & Parikhc (2018) also reported that the changes in phosphatase activity ratios after lime application indicated a short-term impact of lime on the enzymatic component of P cycling, which could translate to longer-term changes in organic P mineralization and available P. Grove & Sumner (1985) also found that the soil Al polymers could precipitates soil elements (P, Zn, Mg) in over-limed soils. Therefore, in acid soils, the soil AP concentration should be evaluated when using lime as soil amendments, confirming whether phosphorus fertilizer is also needed besides lime application in acid soil (Perrott & Mansell, 1989).

Compared with the lime application, biochar could also increase pH and reduce soil exchange Al concentration, suggesting that it was also an alternative method in improving acid soils for legume green manures. Biochar is derived from the thermal decomposition of plant biomass under anaerobic environment (Blanco-Canqui, 2017). It is commonly reported that biochar benefits are related to an increase of CEC, sorption capacity, contribution of nutrients and creating a favourable environment for microbial growing (Blanco-Canqui, 2017). A decrease of aluminum toxicity induced by biochar application could increase soil pH and the concentrations of soil available nutrients, resulting in increased plant growth in several studies (Jha et al., 2016; Lin et al., 2018; Qian, Chen & Hu, 2013). In the current study, overall biochar application increased the concentrations of soil AP, AN and AK, thus benefiting plant nutrient uptake. Pandit et al. (2018) also reported that biochar addition could increase the contents of maize potassium and soil AP. However, Prapagdee & Tawinteung (2017) reported that the increased biochar application significantly increased the concentrations of soil TN and extractable K but did not affect the amount of AP. The variations of soil AP in responding to biochar application might be attributed to variations of the plant materials used for biochar and pyrolysis conditions in these studies (Hartley, Riby & Waterson, 2016). A study with nine types of biochar (one natural woody biochar and eight manufactured plant derived biochars) has shown that biochars were able to bring available P into soils, but the amount and form of available P was dependent on biochar types (Zhang et al., 2016). Cheng et al. (2018) also reported that soil dissolved organic carbon and available P increased in soils applied with wheat straw biochar produced at high pyrolysis temperature. However, in this study, due to the significant interactions between biochar and lime applications on nutrient uptakes, the highest P and K uptake was observed at B1 under L2 condition. Overall, the results indicated that addition of biochar from *S. tuberosum* vine could improve the acid soil fertility.

Both biochar and lime could increase the contents of MBC and MBN and their ratio in soils, indicating that the increased soil pH and decreased soil exchangeable Al concentration improved the soil microbe activities. Quilliam et al. (2013) also reported that biochar addition would change soil physicochemical properties, alter soil microbial activity and structure, and ultimately affect soil-plant-microbe interactions. Nguyen et al. (2018) also reported that the acclimation of soil bacteria on receiving repeated biochar amendment leaded to similar bacterial diversity and community structure among 9-years old applied biochar, repeated biochar treatments and control. Though we had not measured the soil microbe communities, the soil microbe species in limed and biochar added soils might also be different, resulting in their different influences on soil nutrient cycles. When biochar is added into soil, its surface will be coated with organic compounds, which may result in improved nutrient retention and create a more optimal habitat for soil microorganisms (McLauchlan, Hobbie & Post, 2006). In the current study, lime and biochar application also increased the urease and dehydrogenase activities, mainly attributing to the changes of soil physiochemical properties and soil microbe communities induced by lime and biochar application. Higher organic carbon in biochar might partly result in higher enzymatic activities (Rao & Ghai, 1985). Higher soil urease activity would benefit the hydrolysis of soil nitrogen, whereas higher dehydrogenase activity represented improved soil redox processes in limed and biochar added soils (Bailey et al., 2011).

### Effect of treatments on plant growth and nutrient uptake

As a green manure, obtaining optimum aboveground biomass is the prerequisite to achieve high fertilizer efficiency (Edmeades, 2003). Acid soils severely limited the growth of *P. calcaltus*. The shoot biomass was 4.33 g pot^−1^ in un-limed soil, which increased by 20.09% to 5.20 g pot^−1^ in soils applied with 1.5 g kg^−1^ lime. Biochar addition significantly increased the biomass only under high lime rate (1.5 g kg^−1^) but not at low lime rates or control. It suggested that lime might be better in improving the growth of *P. calcaltus* in the tested acid soil when compared with biochar addition. Using global-scale meta-analysis, Jeffery et al. (2017) found that biochar application particularly benefited agriculture in acidic soils in the tropics, showing no universal yield-stimulating effects. Based on a study in a moderately acidic Inceptisol, Pandit et al. (2018) also reported that the increased maize biomass production in acid soil was directly benefited from the alleviation of nutrient stresses upon biochar addition but not from liming or soil pH. This implied that nutrient stress usually existed in low pH soils and only when the soil pH reached a relative higher level could the biochar improve the plant growth further when both lime and biochar were applied.

When compared with the liming, the changes of soil exchange Al concentrations were much smaller among biochar rates, particularly at L0 (reduced by 22% averaged across B1 and B2) and L1 (reduced by 19%), where the soil exchange Al concentrations were all larger than 1.64 cmol kg^−1^. This might be the main reason why no further growth improvement could be observed under low lime rates. Under high lime rate (L2), the soil exchangeable Al concentrations reduced to lower than 1.11 cmol kg^−1^, with its concentration reduced by 27.76% at B1 and 68.68% at B2, which might alleviate the limitations of Al^3+^ on plant growth. Surprisingly, the highest plant biomass was observed at B1 but not at B2 under high lime rate (L2), and the plant P concentrations was also higher at B1 than at B2 under L2. One possible reason might be that high biochar addition (B2) under high lime rate (L2) might further enlarge the nutrients imbalance, which might offset the positive influence on plant growth induced from reducing soil exchangeable Al concentrations. For example, in the current study, biochar application significantly increased the ratio between the concentrations of AK and AP, mainly attributed to the increased potassium release into soil through biochar. Limwikran et al. (2018) also reported that biochar addition could release large amount of K^+^ into soils, which might be exchanged with the Ca^2+^ in soil, causing alteration of soil ion ratios, and finally leading to lower yields (Loide, 2004). In a 3-year mesocosm experiment on sandy and silty soils, Borchard et al. (2014) found that increasing the application rate of charcoal resulted in decreased plant biomass in the second and third year of the experiment, likely as a result of nutrient imbalances and N-immobilization. Therefore, when using biochar or lime to improve acid soil fertility, their application rates should be evaluated according to their efficiency in improving final crop productivity.

In the current study, high biochar addition significantly increased the leaf net photosynthesis rate with transpiration rate unchanged under all lime rates. This was inconsistent with the responses of biomass to biochar application in un-limed and low limed soils. One possible reason might be that the increase of photosynthesis during daytime might be offset by relatively higher respiration rate during evening in biochar added soils (De Block & Van Lijsebettens, 2011).

In the current study, no nodule was observed in un-limed soil when no or low biochar was added, suggesting that higher soil exchangeable Al concentrations and low pH affected the affinity between Rhizobium and plant root (Foy, 1988; Roesner, Fettell & Brockwell, 2005). Lime and biochar application increased the nodule number and nodule dry weight, mainly attributed to the reduced exchangeable Al concentration and increased pH. Under same lime rate, biochar application further increased the nodule number and dry weight, which might be attributed to the further reduce of soil exchange Al concentration at B1 and B2. However, long-term field studies with a variety of biochar rates are needed to clarify the influence of biochar application on nodulation abilities of *P. calcaltus* in acid soils, finally providing agronomic management decisions in planting legume green manures in acid soils with varying soil exchangeable Al concentrations (Quilliam, DeLuca & Jones, 2013)

Nutrient content in green manure is another factor affecting the green manure efficiency. Generally, more nutrients released into the soil will benefit more on soil fertility. In the current study, lime application increased the plant TN concentration but not TP and TK concentrations, whereas biochar application at B2 increased plant TP concentrations at L0 and L1. When the total nutrient uptake was considered, overall lime and biochar application increased the N and P uptake, suggesting that both lime and biochar application could improve nutrient uptake by *P. calcaltus,* being attributed to the improvement of soil nutrient availabilities. Correlation analysis also indicated that the nutrient (N,P,K) uptake was positively correlated with soil pH and negatively correlated with soil exchangeable Al concentration. In tropical clay soil in Zimbabwe, application of sludge biochar significantly improved the maize growth, biomass production and nutrient uptake when compared to the unamended control (Gwenzi et al., 2016). A pot experiment with a Ferralsol also has shown that biochar application improved the retention of water and nutrients by the soil and thereby uptake of water and nutrients by the plants (Agegnehu et al., 2015). Compared with the biochar, the effects of lime application on plant nutrient uptake have been paid more attention to many acid soils. Though lime could increase nutrient uptake in most circumstance (Caires, Barth & Garbuio, 2006; Haling et al., 2010), results from some studies have shown that overliming would tend to reduce uptake of P and K as well as other micro minerals such as Zn, Cu, Mn, and Fe (Fageria, Zimmermann & Baligar, 1995). In this study, the highest lime rate reached 1,697 kg ha^−1^, which was much lower when compared with other studies (Caires, Barth & Garbuio, 2006; Hodge & Lewis, 1994). However, sole liming might enhance the risk of reducing nutrient uptake as well as altering soil physicochemical properties (Nunes et al., 2017), finally reducing its efficiency in improving soil fertility. In the current study, higher shoot biomass at B1 than at B2 under L2 condition suggested that biochar application incorporated with reducing amount of lime could achieve optimum improvement for *P. calcaltus* growing in acid soils.

Both biochar and lime significantly reduced the concentrations of aluminum in *P. calcaltus*, particularly under un-limed and low lime rate conditions. This was mainly attributed to the reduced soil exchangeable Al in lime and biochar added soils. However, under high lime rate (1,697 kg ha^−1^), no further reduction could be observed in plant aluminum content when biochar was added, and a higher amount of aluminum uptake was also observed in biochar added soils, though soil exchange Al was significantly reduced. This implied that *P. calcaltus* might tolerate or need certain amount of aluminum in soils to grow normally. In a review, Bojorquez-Quintal et al. (2017) also concluded that Al^3+^ could have a beneficial or toxic effect, depending on its concentration, chemical form, and the plant species and growing conditions. This might also explain why higher shoot biomass was observed at B1 but not at B2 under high lime rate (L2), where the soil exchangeable Al concentration reduced to 0.33 cmol kg^−1^ when compared to the 0.75 cmol kg^−1^ at B1.

## Conclusions

The growth of *P. calcaltus* was severely limited in acid soils. Both lime and biochar application could reduce soil exchangeable Al concentrations and increase soil pH, improving soil nutrient availability and microbe activities, plant nutrient uptake, nodulation, and plant growth. Compared with liming, application of sole biochar could not achieve higher shoot biomass. High lime rate (1.5 g kg^−1^; equaling 1,697 kg ha^−1^) incorporated with low biochar rate (5 g biochar kg^−1^; equaling 5,659 kg ha^−1^) could obtain higher shoot biomass, nutrient uptake, and nodule number when compared with high lime rate and high biochar rate. Long term field experiment is still needed to clarify the lime and biochar rates associated with planting *P. calcaltus* in specific acid soils, providing appropriate agronomic management decisions.

##  Supplemental Information

10.7717/peerj.6346/supp-1 Data S1 Raw data of the manuscript Data of all parameters with four replicates.

## Abbreviations

AP: available phosphorus
AN: alkali dispelled nitrogen
AK: available potassium
MBC: microbial biomass carbon
MBN: microbial biomass nitrogen
TOC: total organic carbon
TN: total nitrogen
TP: total phosphorus
TK: total potassium
CEC: soil cation exchange capacity
TTC: triphenyltetrazolium chloride

## Additional Information and Declarations

### Data Availability

The following information was supplied regarding data availability: Raw data are available in the Supplemental Files.
